# Enhanced Resorption of Liposomal Packed Vitamin C Monitored by Ultrasound

**DOI:** 10.3390/jcm9061616

**Published:** 2020-05-26

**Authors:** Lukas Prantl, Andreas Eigenberger, Sebastian Gehmert, Silke Haerteis, Thiha Aung, Reinhard Rachel, Ernst Michael Jung, Oliver Felthaus

**Affiliations:** 1 Department of Plastic, Hand and Reconstructive Surgery, University Hospital Regensburg, Franz-Josef-Strauss-Allee 11, 93053 Regensburg, Germany; Andreas.Eigenberger@ukr.de (A.E.); Thiha.Aung@ukr.de (T.A.); Oliver.Felthaus@ukr.de (O.F.); 2 Faculty of Mechanical Engineering, Ostbayerische Technische Hochschule Regensburg, Galgenbergstr. 30, 93053 Regensburg, Germany; 3 Department of Orthopaedics, University Children’s Hospital, Spitalstrasse 33, 4056 Basel, Switzerland; s.gehmert@gmail.com; 4 Institute for Molecular and Cellular Anatomy, University of Regensburg, Universitätsstraße 31, 93053 Regensburg, Germany; Silke.Haerteis@vkl.uni-regensburg.de; 5 Centre for Electron Microscopy, Faculty of Biology and Preclinical Medicine, University of Regensburg, Universitätsstraße 31, 93053 Regensburg, Germany; reinhard.rachel@biologie.uni-regensburg.de; 6 Ultrasound Center, Department of Radiology, University Hospital Regensburg, Franz-Josef-Strauss-Allee 11, 93053 Regensburg, Germany; Ernst-Michael.Jung@ukr.de

**Keywords:** vitamin C, immune system, liposomal packing, sonography, enhanced resorption

## Abstract

Vitamin C is an essential nutrient for humans and is involved in a plethora of health-related functions. Several studies have shown a connection between vitamin C intake and an improved resistance to infections that involves the immune system. However, the body cannot store vitamin C and both the elevated oral intake, and the intravenous application have certain disadvantages. In this study, we wanted to show a new formulation for the liposomal packaging of vitamin C. Using freeze etching electron microscopy, we show the formed liposomes. With a novel approach of post-processing procedures of real-time sonography that combines enhancement effects by contrast-like ultrasound with a transducer, we wanted to demonstrate the elevated intestinal vitamin C resorption on four participants. With the method presented in this study, it is possible to make use of the liposomal packaging of vitamin C with simple household materials and equipment for intake elevation. For the first time, we show the enhanced resorption of ingested liposomes using microbubble enhanced ultrasound imaging.

## 1. Introduction

Vitamin C is ubiquitous in nature, particularly in fruits and vegetables, with oral ingestion as the primary route of administration in humans, since most vertebrates cannot synthesize ascorbic acid [1]. L-gulonolactone oxidase catalyzes the final step in the biosynthesis of ascorbic acid, but is inactive in primates due to evolutionary conserved deletions. Vitamin C (ascorbic acid) functions as an essential water-soluble electron donor by donating an electron to a substrate. The reduced form of vitamin C is ascorbate, which acts as an antioxidant, and is required as a cofactor for various biological reactions [2]. Moreover, several studies have shown that ascorbate modulates vasorelaxation and regulates the activity of NADPH oxidases involved in inflammatory gene response [3]. Most prominently, vitamin C is known for its ability to prevent and cure scurvy, which appears clinically when the plasma level is below 11 µmol/L resulting from a continuous intake of less than 10 mg/day [4]. Interestingly, individuals with scurvy are highly susceptible to potentially fatal infections, such as pneumonia [5], which links vitamin C to the immune system and immune cell function [6,7]. Furthermore, vitamin C also has an influence on the susceptibility and the duration of colds [8]. Animal studies imply a broader role for vitamin C in the immune response to infections, including pneumonia and sepsis [9,10,11]. Moreover, vitamin C has been shown to protect broiler chicks against an avian coronavirus [12,13,14]. In addition to this, vitamin C was reported to increase the resistance to infections by a coronavirus of chick embryo tracheal organ cultures 40 years ago [15]. Additionally, under vitamin C shortage, influenza leads to more severe lung damage [16] and an influenza infection caused a decrease in vitamin C concentration in bronchoalveolar lavage fluid in mice [17]. Many infections cause the release of reactive oxygen species (ROS) by activated phagocytes [5], which link the antioxidant properties of vitamin C and the immune response. In addition, ROS are important factors involved in mitochondrial damage, which can cause the loss of function of enzymes in the electron transfer system and/or cell death [18]. Studies in critically ill septic patients suggest a problem in cellular respiration rather than in oxygen delivery since oxygen levels in tissue were observed to be elevated while the oxygen consumption was decreased [19]. Thus, antioxidants and antioxidant enzymes are required to reduce oxidative stress during a viral infection or critical illness.

With an oral intake of approximately 200 mg/day of vitamin C in healthy people, the blood level reaches a value of approximately 70 µmol/L that does not increase despite higher intake [20]. However, the recommended intake for vitamin C is 500 mg/day and, therefore, up to one hundred-fold higher than that for many other vitamins [21], and is still controversially debated [22]. Even if this intake is accomplished, some studies imply benefits of further supplementation [5] and a higher demand may arise from physical or psychological stress [23,24]. However, higher doses of orally applied vitamin C lead to gastrointestinal tract dysfunction. Vitamin C is soluble in water and is available in its anionic form (>99.9%) at neutral pH and only diffuses across the plasma membrane at a slow rate, even in the presence of a concentration gradient. Carrier proteins facilitate the diffusion across membranes but require an electrochemical gradient. However, increasing oral doses are associated with decreasing absorption fraction due to the saturation of the sodium ion dependent active vitamin C transporter (SVCT1) [25]. In contrast, SVCT2 is widely expressed in all organs and ensures the distribution of vitamin C from the blood stream into the cells in order to secure local demands [26,27]. In cancer treatment, it has been shown that high blood levels of vitamin C can be achieved with intravenous administration [28]. However, high blood levels do not necessarily correlate with the concentration in tissue and cells. 

Vitamin C plasma concentration can be increased by oral application of liposomes. Lipid aggregates can prevent vitamin C degradation in the gastrointestinal tract [29,30,31,32]. Davis et al. showed that the oral application of 4 g of vitamin C encapsulated in liposomes increased the plasma concentration up to C_max_ (about 200 µM) after 3 hours [33]. Liposomes composed of phosphatidylcholines are an important component of a balanced diet [32]. Despite cholines and phosphatidylcholines being crucial for a wide range of physiological functions, most of the population’s consumption remains far below the recommendations [34]. Phospholipids are not only an important part of a balanced diet, but also exert a positive impact on inflammation and several diseases [35,36,37]. Willer et al. have shown that phosphatidylcholine can inhibit HIV-1 infected cell growth in vitro, and concluded that formula containing phosphatidylcholines are well tolerated by humans, and might be applicable during early stages of HIV-1 infections in order to reduce the number of virus producing cells [38]. It should be noted that in some medical conditions the supplementation with vitamin C is contraindicated. Patients with hemochromatosis or glucose-6-phosphate dehydrogenase (G-6-PD) deficiency should avoid any vitamin C supplementation, and individuals that take iron chelators should not exceed an intake of vitamin C of more than 200 mg/day [39,40]. Additionally, there are conflicting reports over whether excessive vitamin C intake increases urinary oxalate excretion [41,42,43]. Therefore, people with preexisting kidney impairments should be careful with vitamin C supplementation. Here, we present a new composition for the oral intake of vitamin C together with liposomes that adds to the numerous benefits of vitamin C and phospholipids, enables the oral application of elevated doses for extended periods of time, and causes no intestinal disturbances in healthy children and adults.

## 2. Experimental Section

### 2.1. Liposomal Packaging

A total of 477 g of distilled water, 115 g of Ethanol (98% vol., Weisshaus GmbH, Füssen, Germany), 161 g of ascorbic acid (Golden Peanut GmbH, Garstedt, Germany), and 13 g of MgCl_2_ is mixed in a glass flask and constantly stirred while placed in an ultra-sonic water bath, until all parts of the solution are completely dissolved. Subsequently, 197 g of lecithin (Powder, from Sunflower, IVOVITAL, Hofgeismar, Germany) is added and mixed with a blender using average speed. The mixture is incubated at 4 °C for 24 h and stirred every 6 hours for 4 minutes. Afterwards, the mixture is poured in a glass flask and placed in an ultra-sonic bath for 45 minutes in order to degas the mixture, in which causes foam on the surface area. The foam is discarded and the remaining mixture is placed in an ultra-sonic water bath again for another 45 minutes. During the two ultra-sonic bath treatments, the water temperature might increase, and in that, case should be replaced by cold water since liposomes tend to resolve when exposed to a higher temperature than 37 °C. The mixture is divided into 50 ml portions for oral intake, of which each one consist of 11.0 g of lecithin, 6.4 g of ethanol (98%), 33.5 g of purified water, and 9 g of vitamin C. Noteworthy, all ingredients can be purchased in online shops or in local drug stores.

### 2.2. Ascorbic Acid Detection

A 2,6-Dichloroindophenol sodium salt hydrate (Sigma Aldrich, St. Louis, MO, USA) solution has a deep blue color. Addition of ascorbic acid reduces the blue 2,6-Dichloroindophenol to the colorless aminodiphenol-form. Although this reaction can be initiated by any strong antioxidant, vitamin C is the only candidate for this in our formulation. The reaction of liposomal vitamin C, a control group based on the same concentration of vitamin C, as well as purified water, was tested. The test solution was a 0.01 mM solution of 2,6-dichloroindophenol sodium salt hydrate. Moreover, 0.8 mL liposomal and non-liposomal vitamin C was diluted with 40 mL of water. Subsequently, the diluted vitamin C was added to the 2,6-Dichloroindophenol in 10 µL steps, stirred and afterwards the absorbance was measured photometrically at a wavelength of 600 nm with a multiwell plate reader (VarioScan, Thermo Scientific, Waltham, MA, USA).

### 2.3. Freeze-Etching

About 1.5 µL of the liquid sample was applied onto a gold carrier for freeze-etching (BALTIC preparation, Wetter, Germany) and immediately frozen by plunging it into liquid nitrogen. The carrier was placed onto a sample holder of the freeze-etching device (T:·−183 °C) and loaded into the freeze-etch unit (CFE-50; *p* < 10^−6^ mbar; Cressington, Watford, UK). The frozen sample was fractured with a cold knife (T < −175 °C) and etched for 4 minutes at a temperature of T = −97 °C (sublimation of the surface water of the sample; ’freeze-etching’). Afterwards, the sample was coated with 1 nm Pt/C (Platinum/Carbon; electron beam evaporation) under an angle of 45 °C, and with about 10 nm carbon at 90 °C for the stabilization of the replica. After removal of the samples from the freeze-etch unit, the replicas were floated onto the surface of sulfuric acid (70%) in order to remove the remainder of the organic substance. The replica was washed three times with distilled water and finally collected onto copper grids with a high transparency (Hex600; G2670C, Plano, Wetzlar, Germany). The samples were analyzed and micrographs taken at 80 kV with an EM 902 transmission electron microscope (Zeiss, Oberkochen, Germany), equipped with a 2k × 2k side-entry CMOS camera (Tröndle TRS, Moorenweis, Germany).

### 2.4. Ultrasound Examination of Intestinal Absorption

All examinations were performed by one experienced certified examiner (DEGUM 3), with over 20 years of sonogram experience, using a multi frequency convex transducer (C1-6Mhz) on a high-end machine (LOGIQ-E9 GE Healthcare, Milwaukee, USA). Image storage was done via single images and cine-loops in DICOM (Digital Imaging and Communications in Medicine) and was sent to PACS (Picture Archiving and Communication System) for independent reading.

After informed consent, and in compliance with the Declaration of Helsinki, four healthy volunteers (1 woman, 1 man, and 2 children, the family of the first author) were included in the study and were preconditioned by fasting for 12 h before the test. Prior to the application of the liposomal suspension a high-resolution ultrasound of the abdomen, the stomach to the duodenum and small bowl to the colon, the liver, kidneys and the bladder was performed. This examination was repeated after drinking either purified water or 50 mL (adults) and 25 mL (children), respectively of the mixture with liposomal vitamin C. The whole family has consumed this food supplement twice a week for a long time in combination with a sufficient fluid intake. Ultrasound images were acquired after 15 min, 30 min, 60 min, 90 min, and 120 min. A double mode from B-mode and microbubble enhanced ultrasound (MEU) was used for the documentation of the bubble penetration through the intestinal wall and to visualize vascular changes such as echo enhancement in the mesenteric vessels and the liver vessels. The mechanical index was reduced to lower than 0.16 (MI ≤ 0.16). Hybrid mode refers to post-processing procedures of real-time sonography that combines the method of 1–5MHz (C1-5-D convex probe) contrast enhanced ultrasound (CEUS) (without contrast agent administration) with a 6–9MHz probe (9L-D linear probe) and a matrix 6–15MHz transducer (ML 6–15-D Matrix Array Linear Probe) [44]. A positive vote by the ethics committee of the Regensburg University for the use of ultrasound for the assessment of abdominal organ perfusion in children exists (reference number 14-101-0015). According to the statement of the European Federation of Societies for Ultrasound in Medicine and Biology (EFSUMB), ultrasound is the primary imaging procedure especially in pediatrics [45]. For the quantification of signal intensities ImageJ (Fiji) [46] was used. Statistical significance between signal intensities was assessed using student’s *t*-test. A *p*-value below 0.05 was considered significant.

## 3. Results

### 3.1. Ascorbic Acid Detection

**Figure 1:**
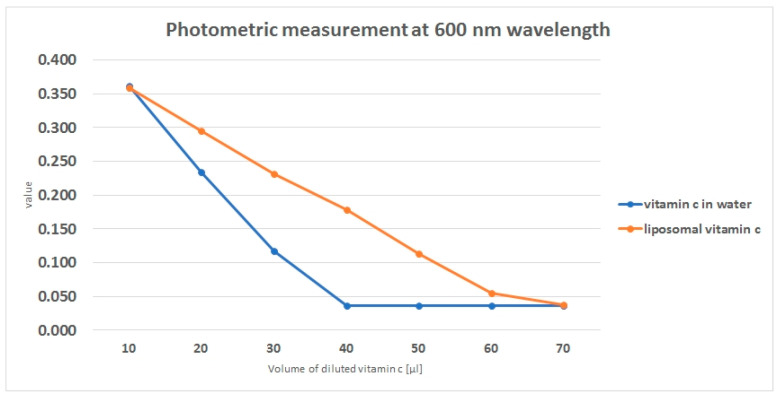
Photometric measurement of 2,6-dichloroindophenol sodium salt hydrate after adding different amounts of liposomal and non-liposomal vitamin C. Absorbance was measured at a wavelength of 600 nm.

To reach a complete decoloration of the 2,6-Dichloroindophenol sodium salt hydrate solution, less than 40 µL of water and vitamin C mixture was enough while 70 µL of liposomal packed vitamin C was needed (Figure 1). An absorbance value below 0.037 was considered as a complete decoloration because of the measured optical density of purified water (0.035 ± 0.0004).

### 3.2. Freeze-Etching

**Figure 2:**
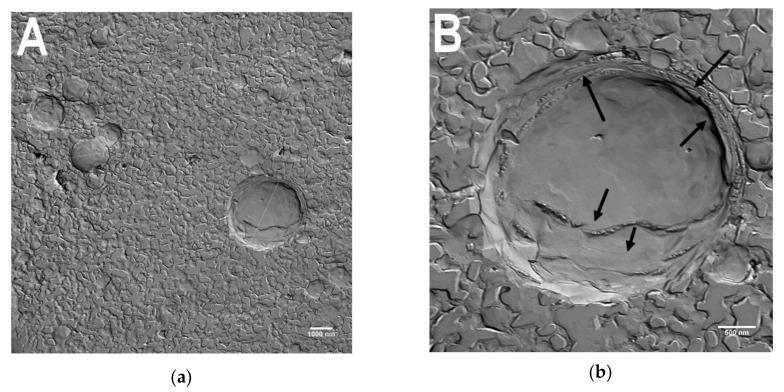
TEM images of freeze-etched liposomes. (**A**) Overview. The diameter of liposomes reaches from ca. 400 nm to ca. 3000 nm. Bar measures 1000 nm. (**B**) Multiple lipid layers can be observed (arrows). Bar measures 500 nm.

Examples of TEM images obtained from the freeze-etched liposomal vitamin C suspension are shown in Figure 2. The liposomes vary in size between 400 and 3000 nm. Arrows in Figure 2B indicate lipid layers.

### 3.3. Ultrasound Examination of Intestinal Absorption

**Figure 3:**
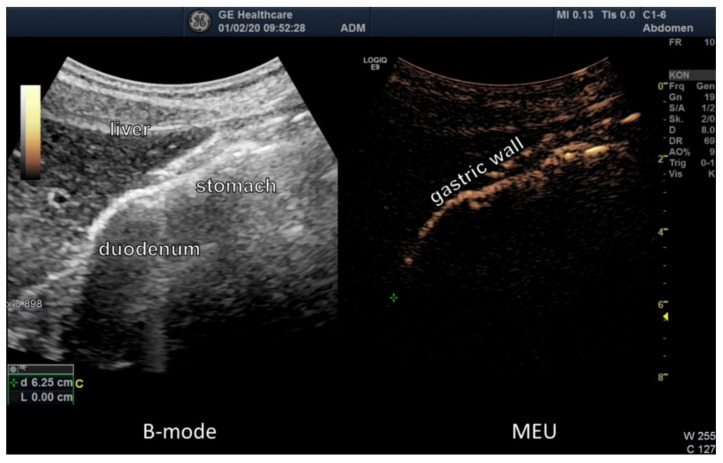
Ultrasound image of the stomach. B-mode after ingestion of the mixture with vitamin C, the substance was immediately detected as an echo amplification by ultrasound in the stomach. The microbubbles (MEU) migrated to the stomach wall and are clearly visible.

Microbubble enhanced ultrasound (MEU) was performed through oral application of 50 mL of the mixture containing 9 g of vitamin C for adults and 25 mL with 4.5 g of vitamin C for children. With the intake of purified water, no signal changes and no penetration trough the intestinal wall could be detected by high-resolution ultrasound. There was no enhanced signal in the mesenteric and liver vessels. After ingestion of the mixture with vitamin C, the substance was immediately detected as an echo amplification by ultrasound in the stomach (Figure 3).

**Figure 4:**
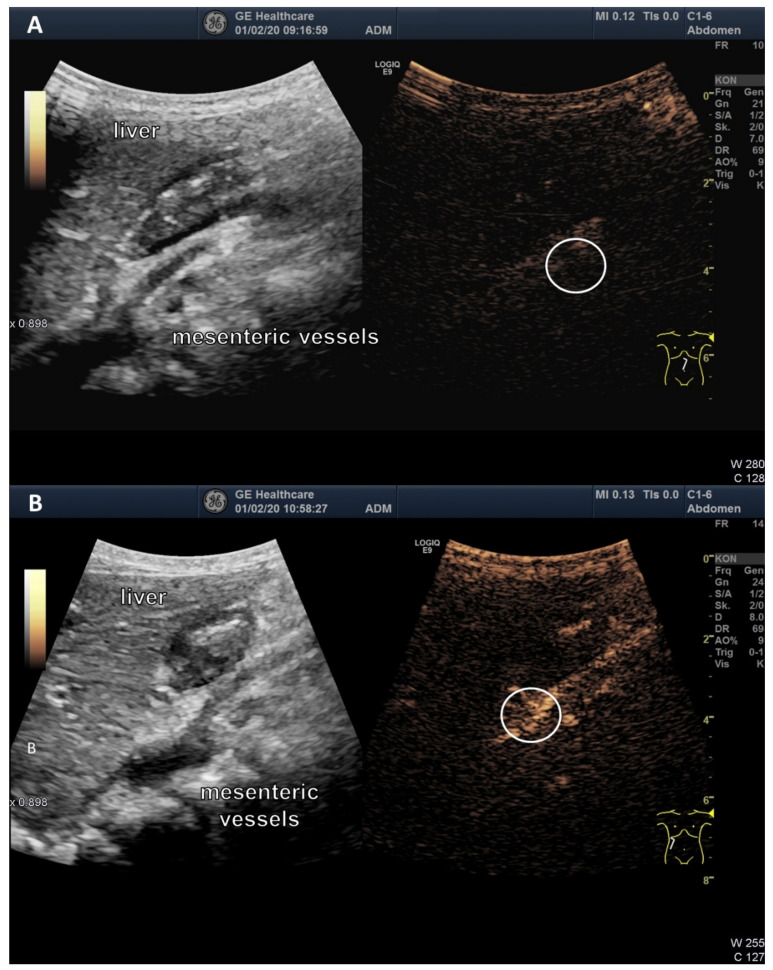
High-resolution ultrasound, multi-frequency C1-6 MHz probe. Abdominal ultrasound images before (**A**) and 90 minutes after oral application of vitamin C formula (**B**) from one volunteer. The circles show the mesenteric vessels. There is a clear microbubble signal indicating the intestinal absorption of liposomes.

**Figure 5:**
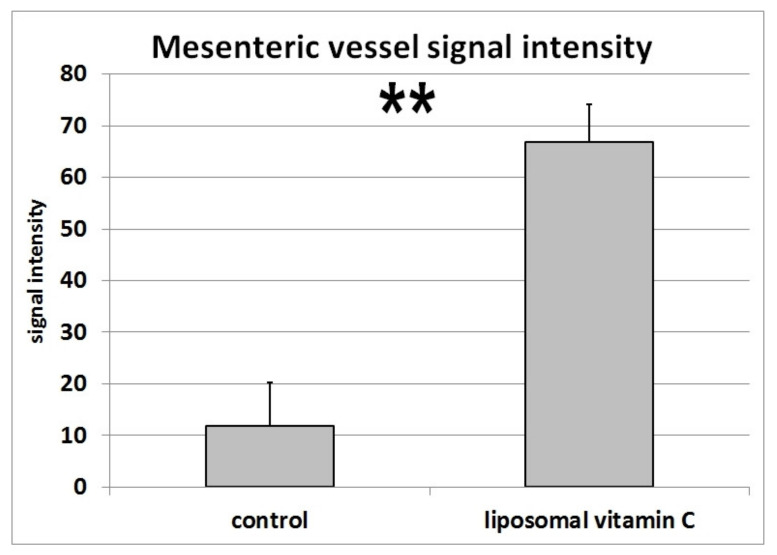
Microbubble signal intensities in mesenteric vessels prior to and following oral vitamin C application for all four patients. Mean and Standard derivations are shown (*p*-value: 0.0013 (**: *p*-value < 0.01))

After only 10 to 15 minutes, an uptake trough the wall of the jejunum could be detected. Some of the microbubbles could be seen in the lumen of the mesenteric vessels up to the portal vein after 30 to 60 min and after 120 min, the signal appeared to be enhanced. Figure 4 shows one patient before and 90 min after liposomal vitamin C uptake with a high signal in the mesenteric vessels, liver and little signal in the stomach as an expression of small remains of the mixture. Signal intensities in mesenteric vessels preceding the oral vitamin C intake, and following the oral vitamin C application, were quantified for all four patients (Figure 5).

**Figure 6:**
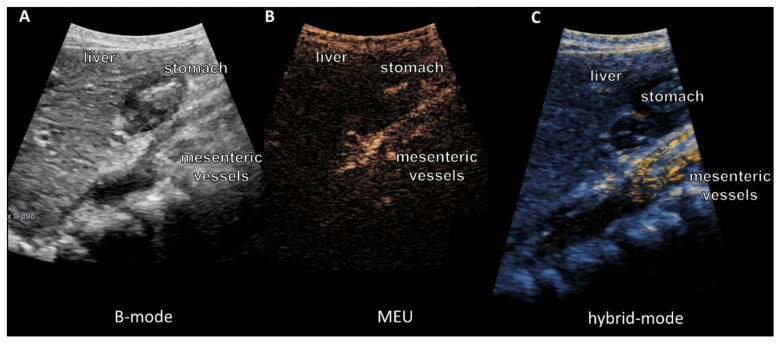
High resolution Ultrasound, multi-frequency C1-6 MHz probe, direct comparison of B-mode (**A**), microbubble enhanced ultrasound (MEU) (**B**), and hybrid mode (**C**) 90 minutes after liposomal vitamin C uptake. (**A**) B-mode after application of the microbubbles with vitamin C (**B**): enhanced signal in the subtraction image with low MI ≤ 0.16, high signal intraluminal in the stomach-duodenum, mesenteric vessels, and liver corresponding to the enhancement of the MEU. (**C**) Visualization of the microbubbles with the hybrid subtraction mode. Clear enhancement of the bubbles in the intraluminal vessels, stomach/duodenum, and liver.

**Figure 7:**
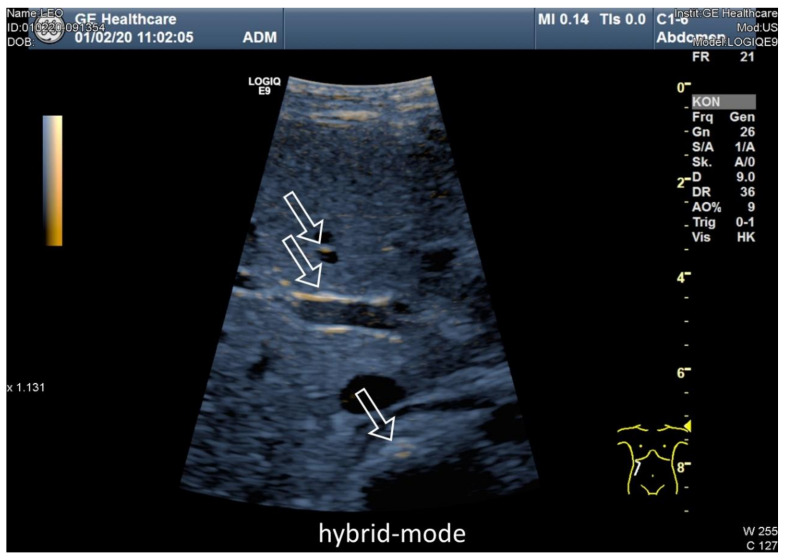
Ultrasound image of liver. Visualization of the microbubbles with the hybrid subtraction mode. Clear enhancement of the bubbles in the larger and smaller liver vessels (arrow).

**Figure 8:**
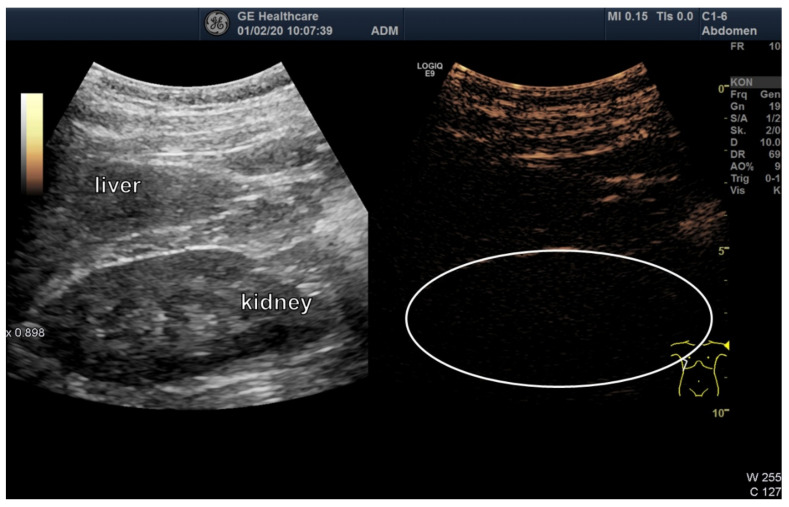
Ultrasound image of kidney. No enhanced signal is detected in the kidney.

Figure 6 shows the direct comparison of B-mode, MEU, and a hybrid mode, demonstrating the uptake of high echo bubbles into the mesenteric vessels. After detection in the mesenteric vessels, the signal was also seen in the liver vessels (Figure 7). No signal was seen in the kidney (Figure 8).

## 4. Discussion

Vitamin C is not only an essential nutrient with antioxidant properties, but also plays an important role for the immune system [5,7,47]. Both the oral application and the intravenous administration have limitations when trying to reach and maintain saturated plasma levels [10,28,32]. However, liposomal packing of vitamin C might be a way to overcome these limitations. It has already been shown that an oral application of liposomal packed vitamin C can raise the plasma level [32]. Although many studies evaluate liposomes and related compounds as a drug delivery system, our method stands out because of the simplicity of the procedure. We investigated the intestinal absorption and the tolerability of liposomal packed vitamin C with a newly revised formula that can be reproduced with simple materials and equipment easily available. Additionally, phospholipids themselves have a beneficial effect on the human body.

Our formula contains liposomes, which are depicted in the electron microscopy images of the freeze-etched sample. These liposomes contain vitamin C, which is indicated by the delayed decoloration of 2,6-Dichloroindophenol sodium salt hydrate when ascorbic acid is combined with lecithin. During ultrasound monitoring, we received a signal from these liposomes that resembles that of ultrasound contrast agents. These are made up of encapsulated microbubbles in suspension and are applied to evaluate the perfusion, to characterize tumors, to assess vesicoureteral reflux and other indications [48,49,50]. The gas core (e.g., sulfur hexafluoride) of these contrast agents is surrounded by a stabilizing shell of phospholipids, albumin, or other material, and enables a higher level of persistence in the circulatory system for several minutes. The bubbles remain strictly intravascular and are not filtered by the glomeruli [51]. The microbubbles scatter the ultrasound beam due to their size and difference in acoustic impedance. We hypothesize that the ultrasound brings the liposomes in oscillation, which causes distinctive echoes when compared to the tissue. We have used a low mechanical index of (MI ≤ 0.16) to preserve the microbubbles and to avoid inducing substantial bio effects. Further investigation in vitro is needed to understand the exact interaction between the ultrasound waves, the mechanical index pulses, and the liposomes.

For the first time, we monitored that the progress of vitamin C loaded liposomes from the stomach to the duodenum and to the distal small bowl using dynamic high-resolution ultrasound. Noteworthy, oral application of the liposomal mixture showed a significant uptake trough the duodenum and jejunum. In addition, 30 to 60 min later, the liposomes were detected in the mesenteric vessels and became a lot more distinct after 90 minutes; we were able to see the signal from the liver vessels. We demonstrated that the liposomes adhered to the intestinal wall, producing an intensified signal and then penetrate through the mucosa [52]. We assume that hypervascularization occurs and that the bubbles can migrate into the mesenteric vessels. It is important to notice that our data originates from a small number of individuals. Especially the results regarding the signal intensity quantification should be interpreted with care due to the limited number of participants. However, our results can be seen as a proof of principle, nevertheless.

For the first time we were able to show changes of the echo signal in the luminal and mural structure of the bowel and the mesenteric vessels. In general, the transabdominal ultrasound is mostly used for the detection and evaluation of gastrointestinal wall and lesions. Liur et al. developed a method of transabdominal ultrasound after oral administration of an echo rich cellulose-based gastric ultrasound contrast agent (TUS-OCCA) [53]. In contrast to the echo rich cellulose-based agent (Huzhou East Medical Devices, Huzhou, Zhejiang, China) that remains in the intestinal lumen, our microbubbles migrated through the intestinal wall and transferred the active substance, in this case ascorbic acid, into the blood circulation and the cells [54]. Of course, the possibilities for liposomal packing and the successive resorption enhancement are not limited to vitamin C. With little adjustments to the procedure, enhanced resorption after liposomal packing might be possible for a large variety of essential nutrients. However, worldwide tragedies like the corona virus pandemic remind us of the importance of the prevention of infections and the strengthening of the immune system. The four volunteers, including the two children aged 9 and 12 years, consume the mixture regularly and can confirm that it is very well tolerated by the intestine and body. However, because of the conflicting results regarding the connection between vitamin C intake and kidney stone formation, people with renal diseases should be careful with vitamin C supplementation.

The exact mechanism of ultrasound on the liposomes and the intestinal resorption remain to be elucidated. The results of this study should be confirmed with a higher number of participants from a randomized sample population. In the future, liposome packing should be evaluated in vitro.

## 5. Conclusions

Our revised formula for liposomal packed vitamin C can be reproduced easily with simple household materials and equipment and can be considered a well-tolerated dietary supplement for healthy individuals. For the first time, we observed the uptake of ingested liposomes following their way from the gut to the mesenterial vessels and liver vessels as a proof of principle via microbubble enhanced ultrasound (MEU) with all the advantages of real time imaging.
